# Comorbidity between physical illness, infection diseases and mental disorders

**DOI:** 10.1192/j.eurpsy.2023.57

**Published:** 2023-07-19

**Authors:** A. Fiorillo

**Affiliations:** University of Campania, Naples, Italy

## Abstract

**Abstract:**

A paradox of the modern world is represented by the increasing rate of comorbidities, although the life expectancy is increasing worldwide, the number of disease-free years is not improving consequently. Physical comorbidities are often overlooked in people with severe mental disorders, although this problem needs to be adequately managed since it is associated with a worse quality of life and a poorer personal and social functioning. In particular, in people with severe mental disorders is very common the contemporary presence of infectious diseases (mainly TBC and HIV) and other physical conditions, which worsen the long-term prognosis.

**Disclosure of Interest:**

None Declared

